# Supplementary material for: MoBiFC: development of a modular bimolecular fluorescence complementation toolkit for the analysis of chloroplast protein–protein interactions
Source: Plant Methods. 2022 May 26;18:69. doi: 10.1186/s13007-022-00902-1 (PMC9134606; doi:10.1186/s13007-022-00902-1)
Supplement: Supplementary file 1 — Additional file 1: Fig. S1. Validation of 3FLAG_nYFP and 3HA_cYFP for BiFC. Fig. S2. Quantification of the BiFC experiments shown in Fig. 2. Fig. S3. Related to Fig. 4, the sensitivity of the BiFC can be adjusted with different FP splits and promoters. Fig. S4. mCHERRY FP fragment fusions do not show YFP fluorescence when expressed alone. Fig. S5. Chloroplastic CFP is unreliable as a reference FP. Fig. S6. Epidermal cells have a high rate of false positive signals. Fig. S7. BiFC in Arabidopsis. Fig. S8. Related to Fig. 5. OEP7-mTRQ levels are proportional to POI-nYFP levels. Fig. S9. Functional test of a chloroplast TurboID module. [file 13007_2022_902_MOESM1_ESM.pdf]

Supplementary Data for Velay et al. "MoBiFC: development of a modular bimolecular fluorescence complementation toolkit for the analysis of chloroplast protein-protein interactions."

Additional File 1. Fig S1-S9

Fig. S1. Validation of 3FLAG_nYFP and 3HA_cYFP for BiFC.
Fig. S2. Quantification of the BiFC experiments shown in Fig. 2.
Fig. S3. Related to Fig. 4, the sensitivity of the BiFC can be adjusted with different FP splits and promoters.
Fig. S4. mCHERRY FP fragment fusions do not show YFP fluorescence when expressed alone.
Fig. S5. Chloroplastic CFP is unreliable as a reference FP.
Fig. S6. Epidermal cells have a high rate of false positive signals.
Fig. S7. BiFC in Arabidopsis.
Fig. S8. Related to Fig. 5. OEP7-mTRQ levels are proportional to POI-nYFP levels.
Fig. S9. Functional test of a chloroplast TurboID module.

Additional file 2. Table S1-S2

Table S1 List of modules in MoBiFC toolkit.
Table S2 List of all constructs generated.

Additional file 3. MoBiFC cloning guide.

Additional file 4. Full plasmid DNA sequences for principal modules.

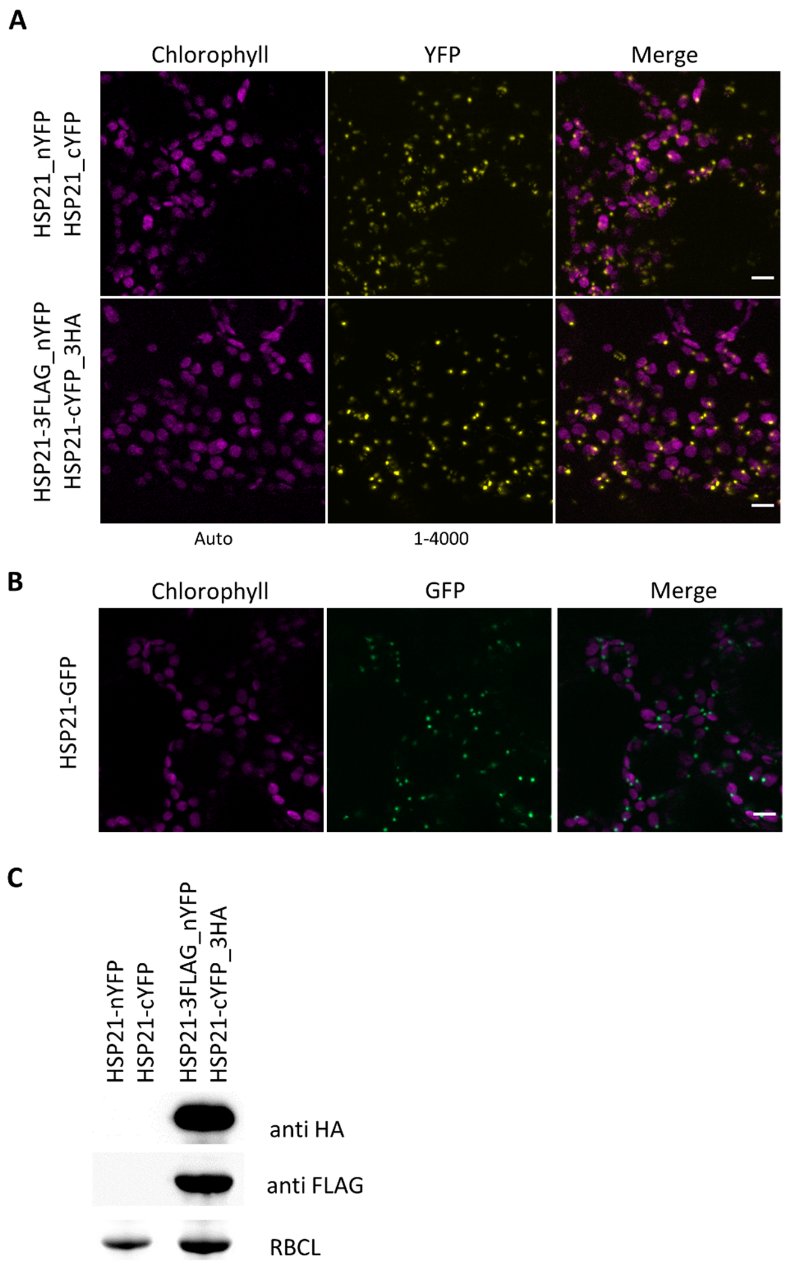

Fig. S1. Validation of 3FLAG_nYFP and cYFP_3HA for BiFC. (A) BiFC assay in *N. benthamiana* mesophyll cells showing the auto-interaction of CTP-SSU chloroplast targeted HSP21 using either untagged YFP fragments (nYFP, cYFP) or epitope-tagged YFP fragments (3FLAG_nYFP, 3HA_CYFP). Scale, 10 µm; histogram levels shown at the bottom of each channel. (B) Localisation of HSP21-GFP in *N. benthamiana* mesophyll cells. Scale, 10 µm. Standard histogram levels are indicated below each channel. (C) Immunoblots with the indicated antibodies on protein samples normalised on a weight basis from the BiFC experiment in panel A. The large subunit of Rubisco (RBCL) was visualised by Sypro fluorescent total protein stain. Modules and plasmids are described in Table S1 and full plasmid sequences are available in additional file 4.

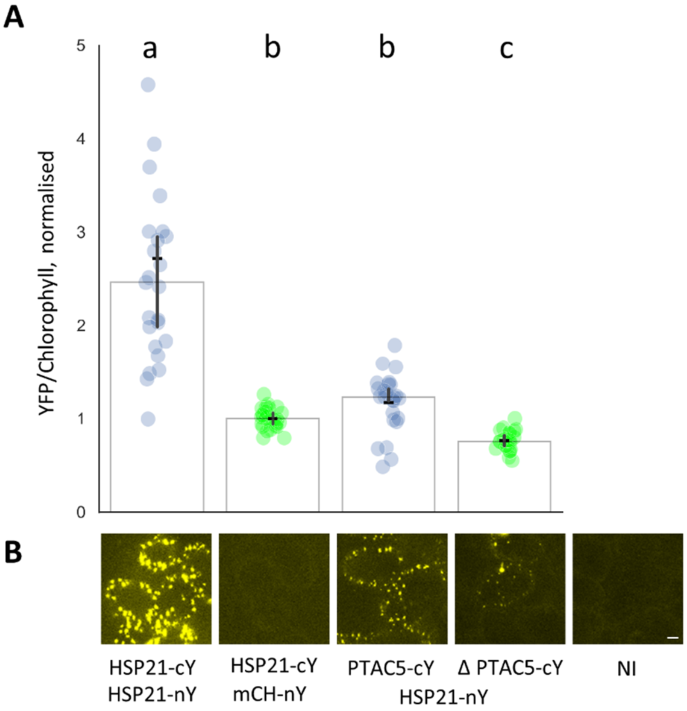

Fig. S2. Quantification of the BiFC experiments shown in Fig. 2. (A) Normalised BiFC signals were calculated as the ratio between total YFP and total chlorophyll fluorescence in transformed cells expressing CFP. The negative control HSP21-cYFP / mCHERRY-nYFP was set to 1. Horizontal line indicates mean and vertical line indicates median +/- 95% confidence interval (n=25-22 transformed cells). Significance was calculated using the Kruskal Wallis test, groups are indicated by lower case letters (P<0.001). (B) YFP channel images from Fig. 4 shown with a lower maximum histogram setting (i.e. saturated) to visualise low intensity signals. Please note that these images are for qualitative comparison and cannot be compared quantitatively due to the saturation. nY, nYFP; cY, cYFP; mCH, mCHERRY; NI, not innoculated.

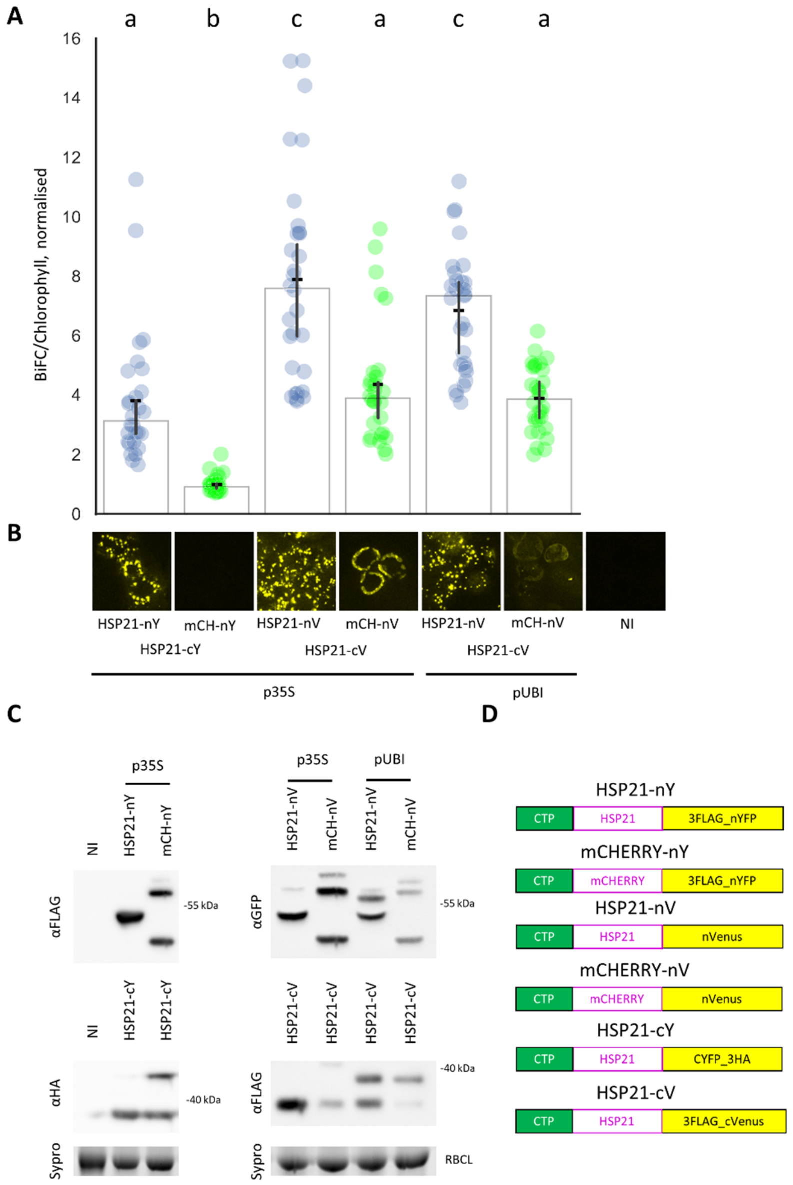

Fig. S3. Related to Fig. 4, the sensitivity of the BiFC can be adjusted with different FP splits and promoters. (A) Quantification of the BiFC experiments shown in Fig. 4. The normalised BiFC signal was calculated as the ratio between total YFP/mVENUS and chlorophyll fluorescence in transformed cells expressing CFP. The negative control HSP21-cYFP / mCHERRY-nYFP was set to 1. Horizontal lines indicate mean and vertical lines indicate median +/- 95% confidence interval (n=28-30 transformed cells). Significance was calculated using the Kruskal Wallis test, groups are indicated by lower case letters (P<0.001). (B) BiFC signal from Fig. 4 shown with a lower maximum histogram setting (i.e saturated) to visualise background signal present in HSP21-cVENUS / mCherry-nVENUS experiments. Please note that these images are for qualitative comparison and cannot be compared quantitatively due to the saturation. (C) Immunoblots with the indicated antibodies on protein samples normalised on a fresh-weight basis from the BiFC experiments in Fig. 4. Detected proteins are indicated above each blot, and sample order corresponds to order in panels A and B. The large subunit of Rubisco (RBCL) was visualised by Sypro fluorescent total protein stain. (D) Summary of proteins detected in panel C. anti-FLAG recognises the FLAG tag in 3FLAG_nYFP (nY) and 3FLAG_cVENUS (cV); anti-GFP recognises 3FLAG_nYFP (nY) and nVENUS (nV); and anti-HA recognises 3HA_cYFP (cY). The large subunit of Rubisco (RBCL) was visualised by Sypro fluorescent total protein stain. mCH, mCHERRY; NI, not innoculated.

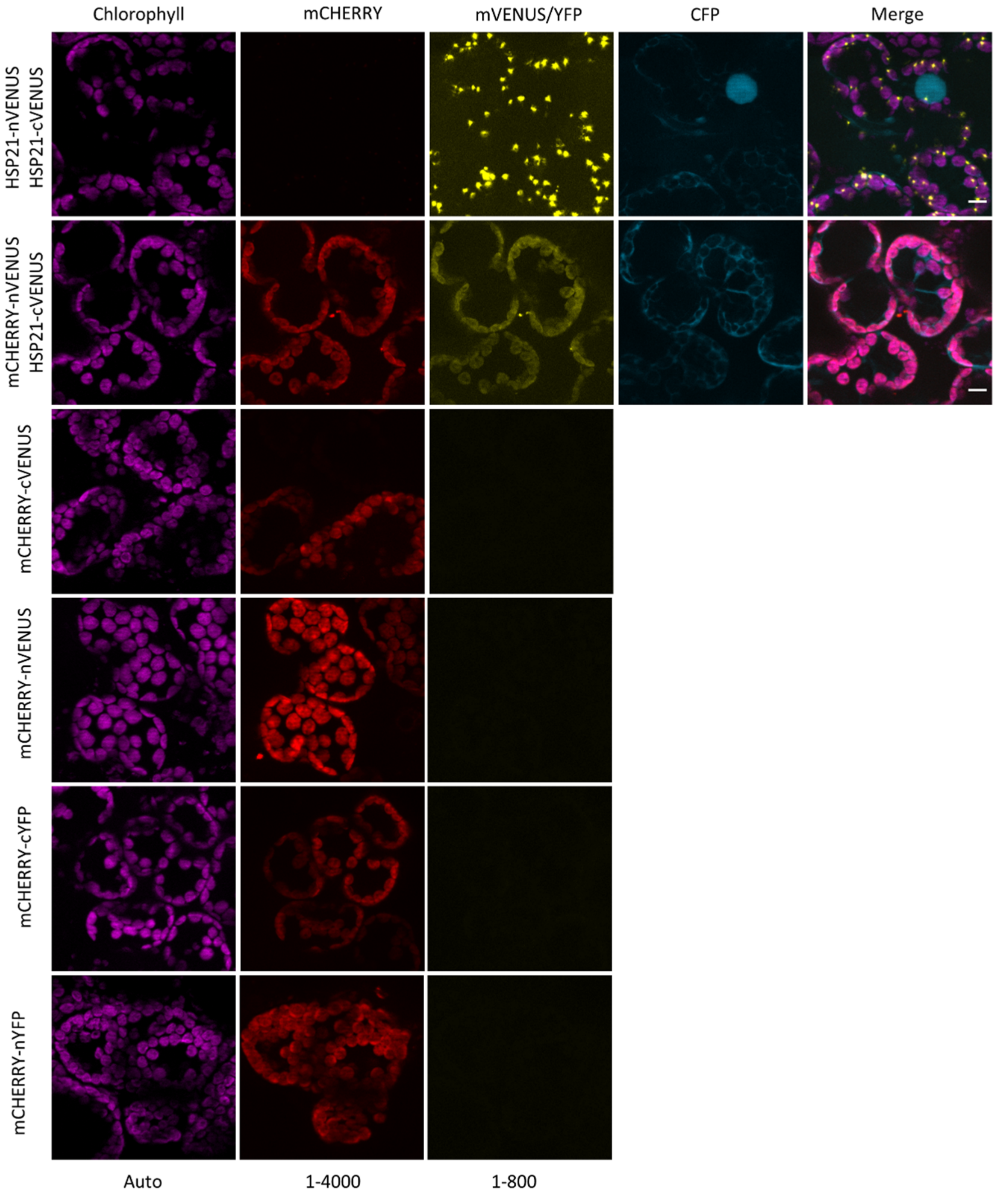

Fig. S4. mCHERRY FP fragment fusions do not show YFP fluorescence when expressed alone. BiFC signals in *N. benthamiana* epidermal cells with the indicated proteins. Scale, 10 μm. Modules and plasmids are described in Table S1 and full plasmid sequences are available in additional file 4.

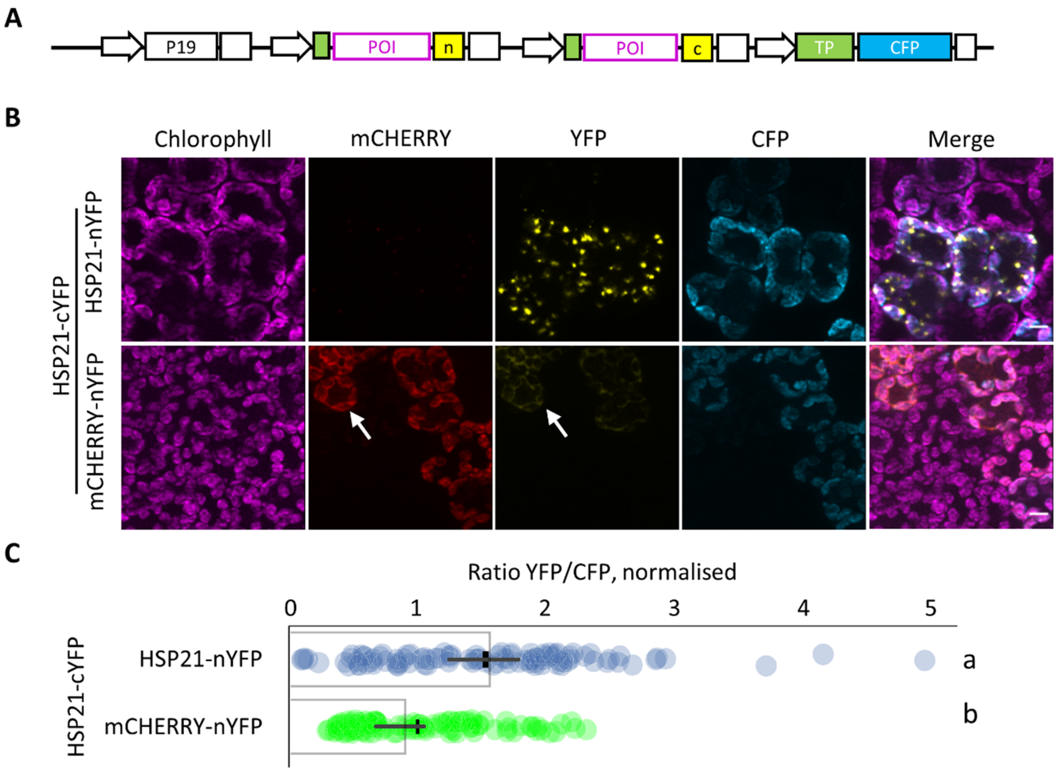

Fig. S5. Chloroplastic CFP is unreliable as a reference FP. (A) Two different HSP21 pairs with the chloroplastic CFP reference FP were used for (B) BiFC assays in *N. benthamiana* mesophyll cells. Scale, 10 µm. Arrows indicate cytosolic signal. (C) Normalised BiFC signals were calculated as the ratio between total YFP and CFP fluorescence. The negative control HSP21-cYFP / mCHERRY-nYFP was set to 1. Vertical line indicates mean and horizontal line indicates median +/- 95% confidence interval (n=100 transformed cells). Significance was calculated using the Kruskal Wallis test, groups are indicated by lower case letters ($P<0.01$). Modules and plasmids are described in Table S1 and full plasmid sequences are available in additional file 4.

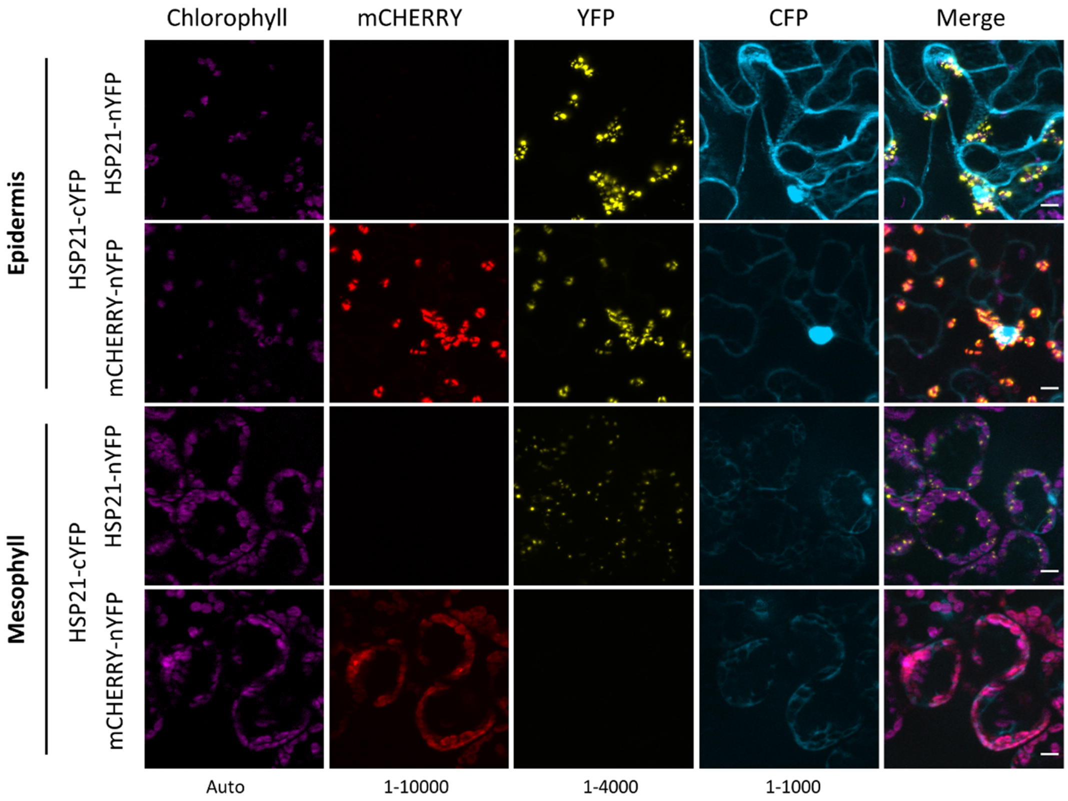

Fig. S6. Epidermal cells have a high rate of false positive signals. HSP21-HSP21 and mCHERRY-HSP21 BiFC assays in *N. benthamiana* epidermal cells (upper two rows) and mesophyll cells (lower two rows). Mesophyll images are of the same region as shown in Fig. 2. Scale, 10 μm.

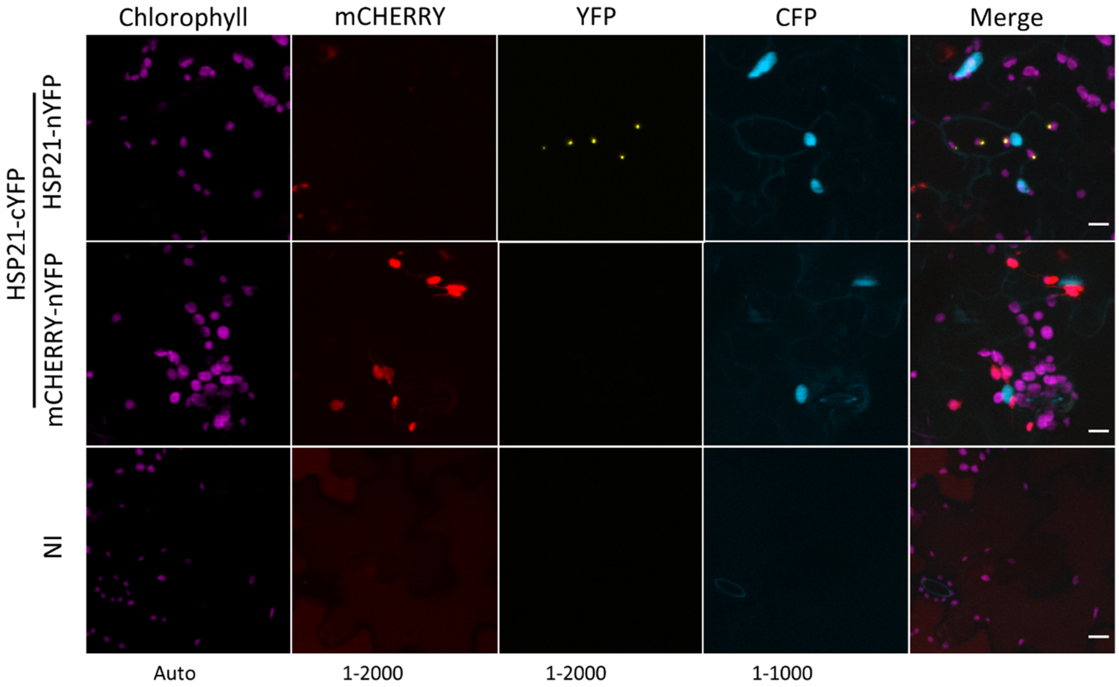

Fig. S7. BiFC in Arabidopsis. HSP21-HSP21 and mCHERRY-HSP21 BiFC assays in *A. thaliana* epidermal cells. No transformed cells were detected in the mesophyll. NI, not inoculated; scale, 10 µm.

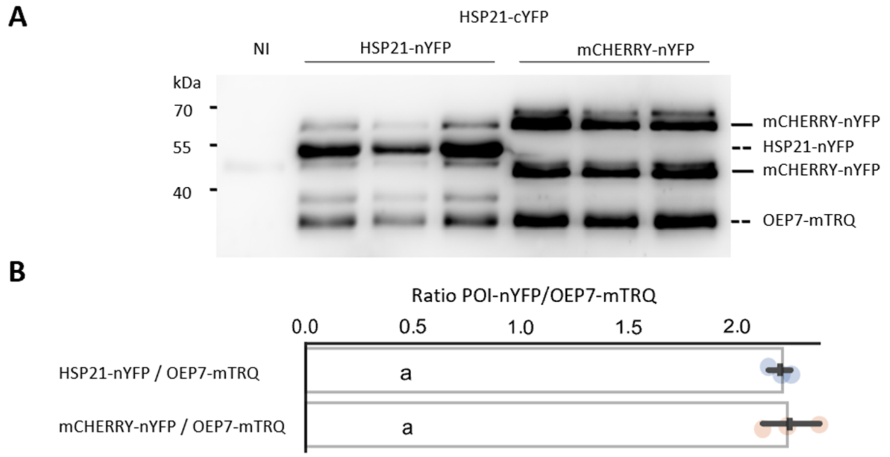

Fig. S8. Related to Fig. 5. OEP7-mTRQ levels are proportional to POI-nYFP levels. (A) Immunoblot with anti-GFP equal quantities of protein from three biological replicates of the two BiFC experiments described in Fig. 5. Detected proteins are indicated to the right, note that anti-GFP recognises mTRQ and nYFP. The large subunit of Rubisco (RBCL) was visualised by Sypro fluorescent total protein stain. (B) Quantification of the ratio between the POI-nYFP and OEP7-mTRQ. Vertical line indicates mean and horizontal line indicates median +/- 95% confidence interval. Significance was calculated using the Kruskal Wallis test, groups are indicated by lower case letters. No significant difference was observed.

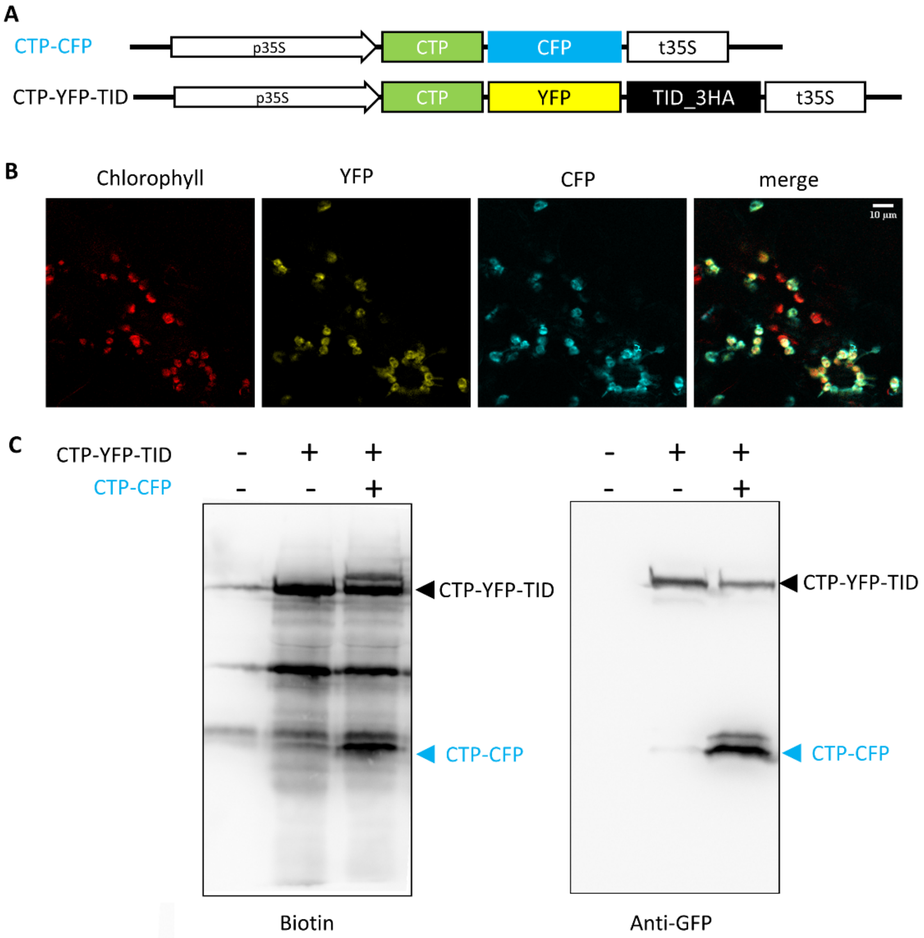

Fig. S9. Functional test of a chloroplast TurboID module. (A) Level 1 modules assembled for testing TurboID (TID). (B) Localisation of CTP-YFP-TID and CTP-CFP in the chloroplasts of co-inoculated *N. benthamiana*. Scale, 10 µm. (C) Detection of biotinylated proteins using streptavidin coupled to horse radish peroxidase and CFP/YFP using anti-GFP antibodies in total protein extracts. Leaf discs from non-inoculated plants, plants inoculated with CTP-YFP-TID only and CTP-YFP-TID + CTP-CFP were incubated with biotin for 1 hr before protein extraction, separation and detection. Biotinylation of the chloroplast localised CFP (CTP-CFP) was observed. Modules and plasmids are described in Table S1 and full plasmid sequences are available in additional file 4.
