# Supplementary material for: MoBiFC: development of a modular bimolecular fluorescence complementation toolkit for the analysis of chloroplast protein–protein interactions
Source: Plant Methods. 2022 May 26;18:69. doi: 10.1186/s13007-022-00902-1 (PMC9134606; doi:10.1186/s13007-022-00902-1)
Supplement: Supplementary file 3 — Additional file 3. MoBiFC cloning guide. [file 13007_2022_902_MOESM3_ESM.pptx]

## Slide 1
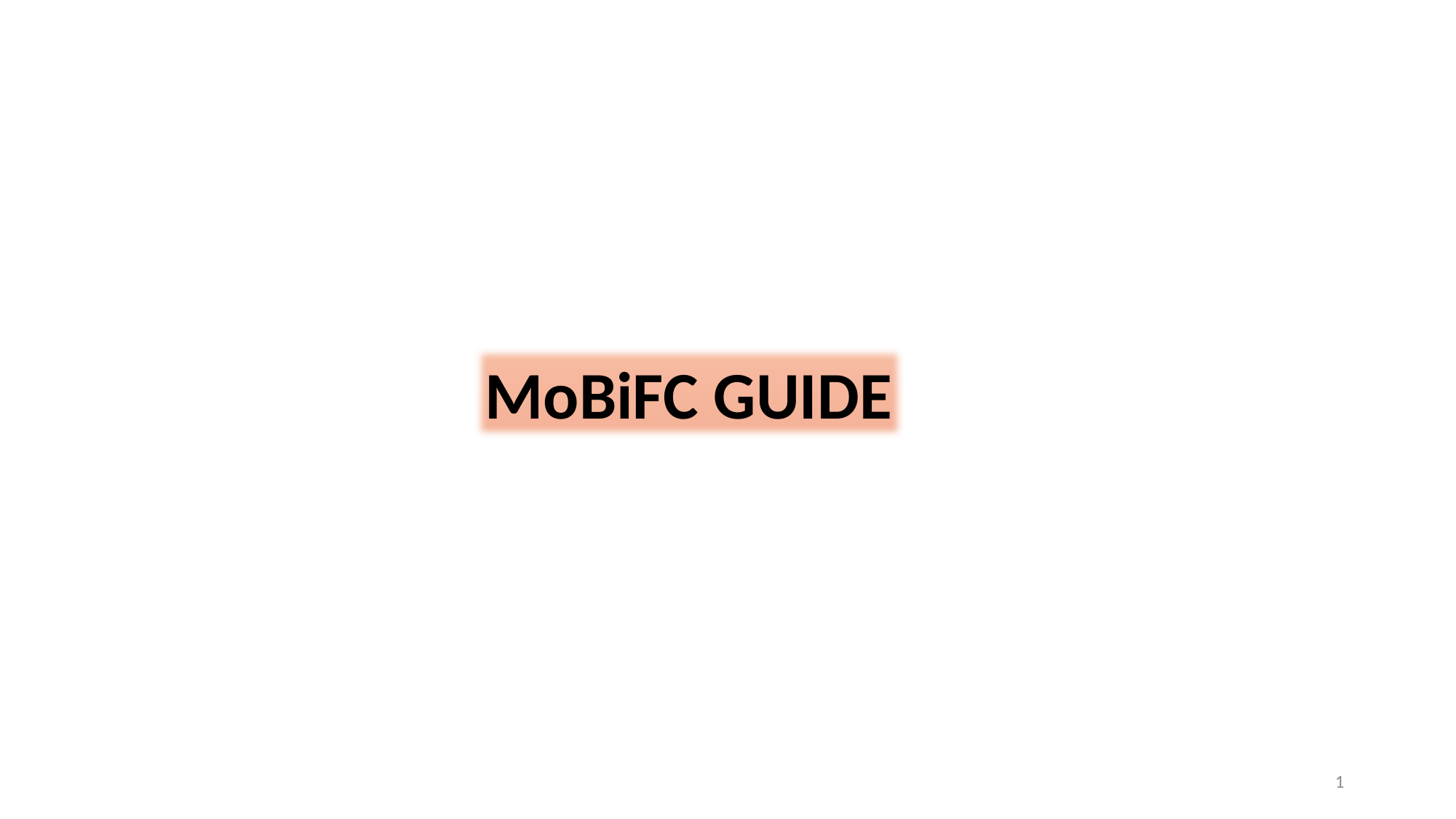

MoBiFC GUIDE
1

## Slide 2
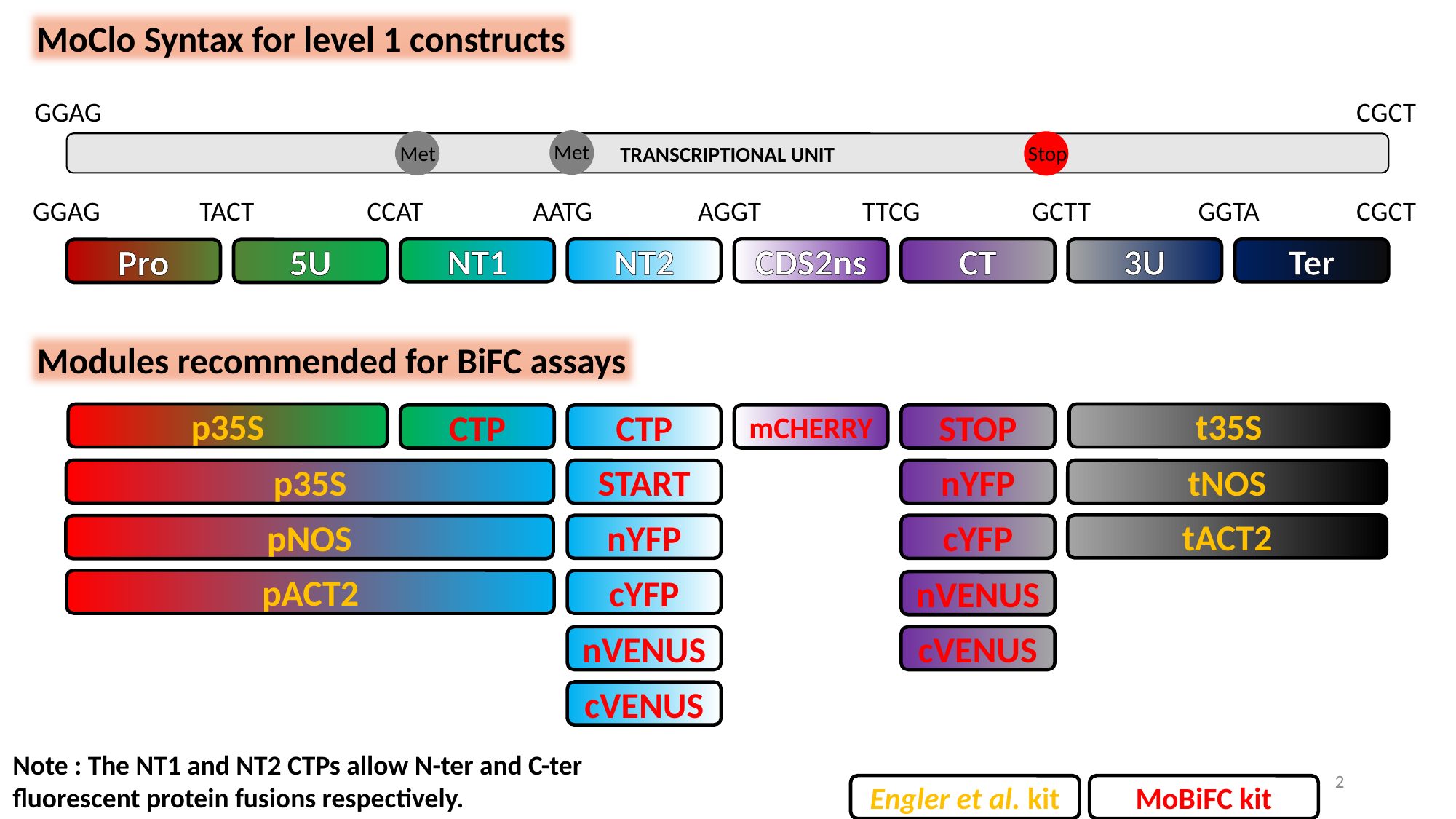

MoClo Syntax for level 1 constructs
GGAG
CGCT
Met
TRANSCRIPTIONAL UNIT
Met
Stop
CGCT
GGAG
TACT
CCAT
AATG
AGGT
TTCG
GCTT
GGTA
NT1
NT2
CDS2ns
CT
3U
Ter
Pro
5U
Modules recommended for BiFC assays
p35S
t35S
CTP
CTP
mCHERRY
STOP
p35S
START
nYFP
tNOS
tACT2
nYFP
cYFP
pNOS
pACT2
cYFP
nVENUS
nVENUS
cVENUS
cVENUS
Note : The NT1 and NT2 CTPs allow N-ter and C-ter fluorescent protein fusions respectively.
2
Engler et al. kit
MoBiFC kit

## Slide 3
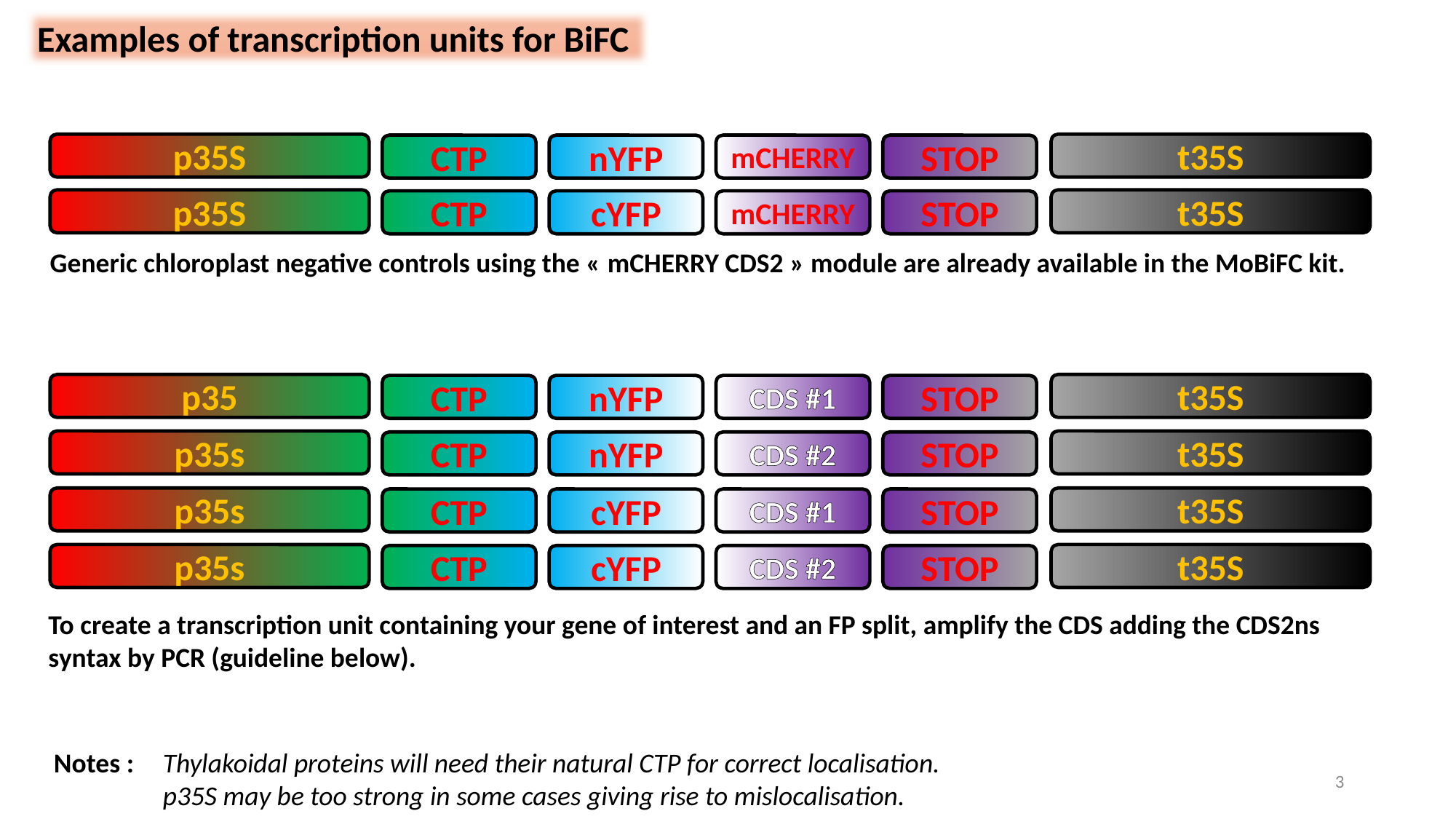

Examples of transcription units for BiFC
p35S
t35S
CTP
nYFP
mCHERRY
STOP
p35S
t35S
CTP
cYFP
mCHERRY
STOP
Generic chloroplast negative controls using the « mCHERRY CDS2 » module are already available in the MoBiFC kit.
p35
t35S
CTP
nYFP
CDS #1
STOP
p35s
t35S
CTP
nYFP
CDS #2
STOP
p35s
t35S
CTP
cYFP
CDS #1
STOP
p35s
t35S
CTP
cYFP
CDS #2
STOP
To create a transcription unit containing your gene of interest and an FP split, amplify the CDS adding the CDS2ns syntax by PCR (guideline below).
Notes : 	Thylakoidal proteins will need their natural CTP for correct localisation.
	p35S may be too strong in some cases giving rise to mislocalisation.
3

## Slide 4
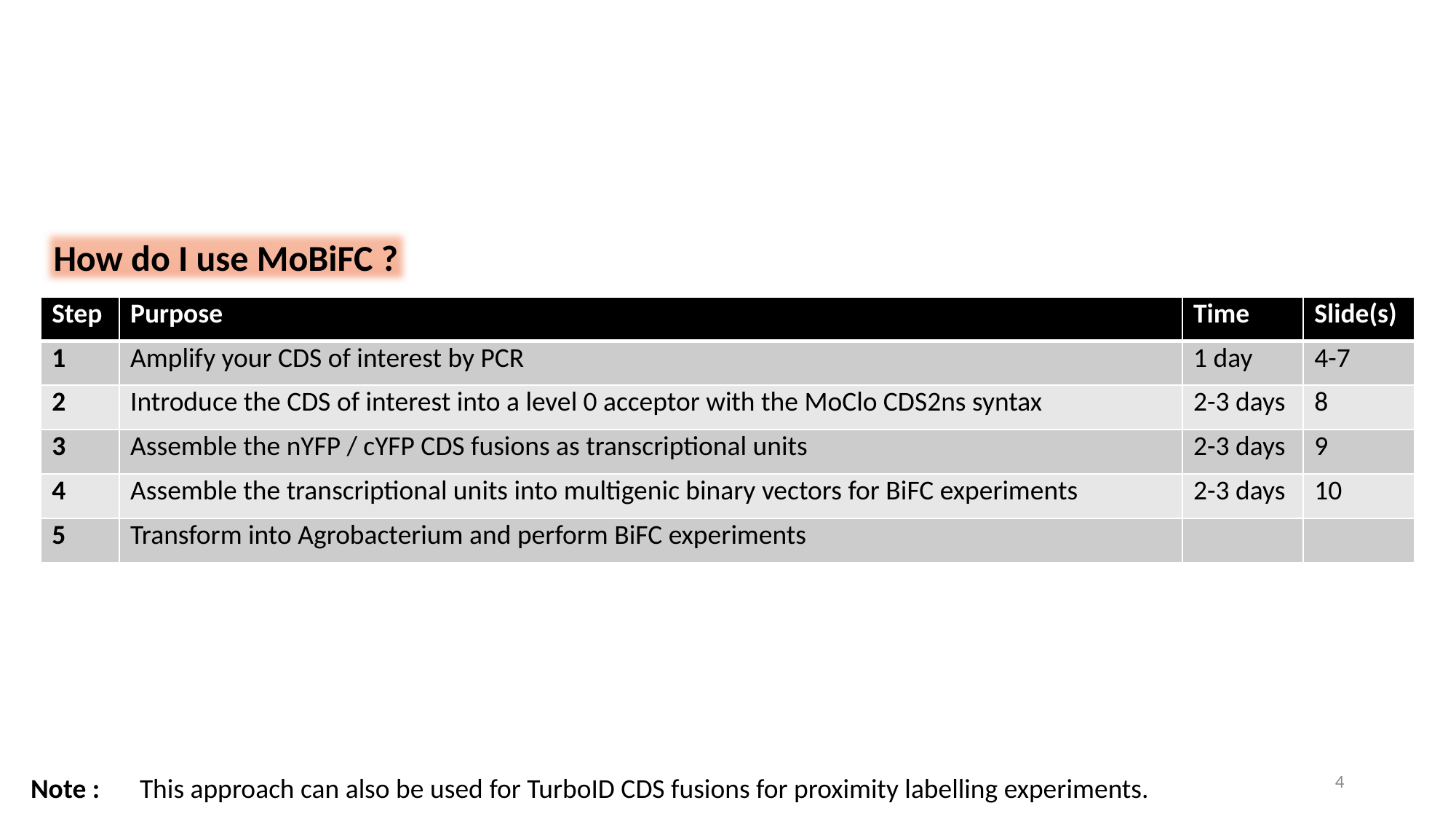

How do I use MoBiFC ?
| Step | Purpose | Time | Slide(s) |
| --- | --- | --- | --- |
| 1 | Amplify your CDS of interest by PCR | 1 day | 4-7 |
| 2 | Introduce the CDS of interest into a level 0 acceptor with the MoClo CDS2ns syntax | 2-3 days | 8 |
| 3 | Assemble the nYFP / cYFP CDS fusions as transcriptional units | 2-3 days | 9 |
| 4 | Assemble the transcriptional units into multigenic binary vectors for BiFC experiments | 2-3 days | 10 |
| 5 | Transform into Agrobacterium and perform BiFC experiments | | |
4
Note : 	This approach can also be used for TurboID CDS fusions for proximity labelling experiments.

## Slide 5
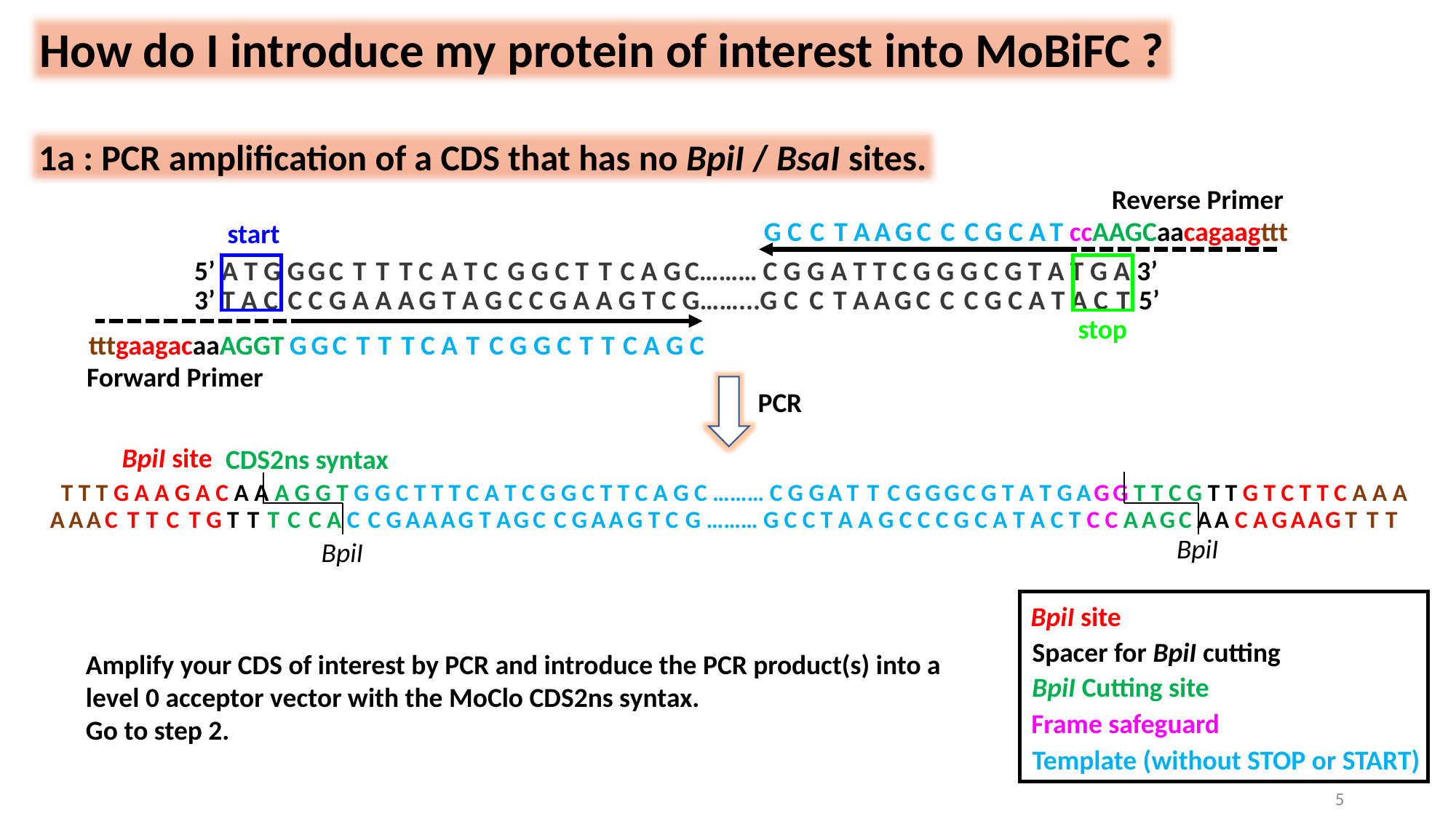

How do I introduce my protein of interest into MoBiFC ?
1a : PCR amplification of a CDS that has no BpiI / BsaI sites.
Reverse Primer
G C C T A A G C C C G C A T ccAAGCaacagaagttt
start
5’ A T G G G C T T T C A T C G G C T T C A G C……… C G G A T T C G G G C G T A T G A 3’
3’ T A C C C G A A A G T A G C C G A A G T C G……...G C C T A A G C C C G C A T A C T 5’
stop
tttgaagacaaAGGT G G C T T T C A T C G G C T T C A G C
Forward Primer
PCR
BpiI site
CDS2ns syntax
T T T G A A G A C A A A G G T G G C T T T C A T C G G C T T C A G C ……… C G G A T T C G G G C G T A T G A G G T T C G T T G T C T T C A A A
A A A C T T C T G T T T C C A C C G A A A G T A G C C G A A G T C G ……… G C C T A A G C C C G C A T A C T C C A A G C A A C A G A A G T T T
BpiI
BpiI
BpiI site
Spacer for BpiI cutting
Amplify your CDS of interest by PCR and introduce the PCR product(s) into a level 0 acceptor vector with the MoClo CDS2ns syntax.
Go to step 2.
BpiI Cutting site
Frame safeguard
Template (without STOP or START)
5

## Slide 6
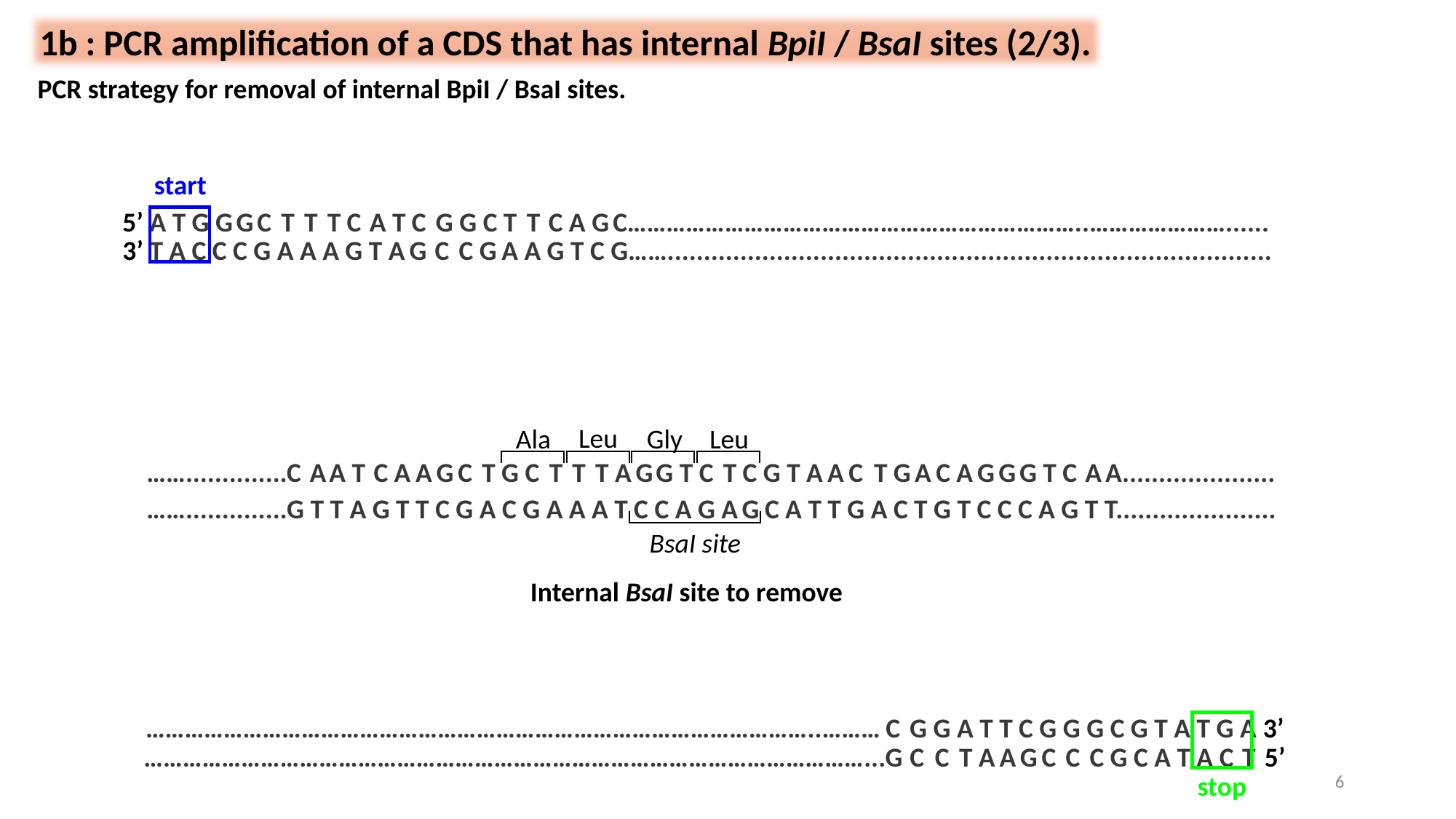

1b : PCR amplification of a CDS that has internal BpiI / BsaI sites (2/3).
PCR strategy for removal of internal BpiI / BsaI sites.
start
5’ A T G G G C T T T C A T C G G C T T C A G C……………………………………………………………..…………………......
3’ T A C C C G A A A G T A G C C G A A G T C G……...................................................................................
Leu
Leu
Ala
Gly
……..............C A A T C A A G C T G C T T T A G G T C T C G T A A C T G A C A G G G T C A A.....................
……..............G T T A G T T C G A C G A A A T C C A G A G C A T T G A C T G T C C C A G T T......................
BsaI site
Internal BsaI site to remove
…………………………………………………………………………………………..……… C G G A T T C G G G C G T A T G A 3’
…………………………………………………………………………………………………...G C C T A A G C C C G C A T A C T 5’
6
stop

## Slide 7
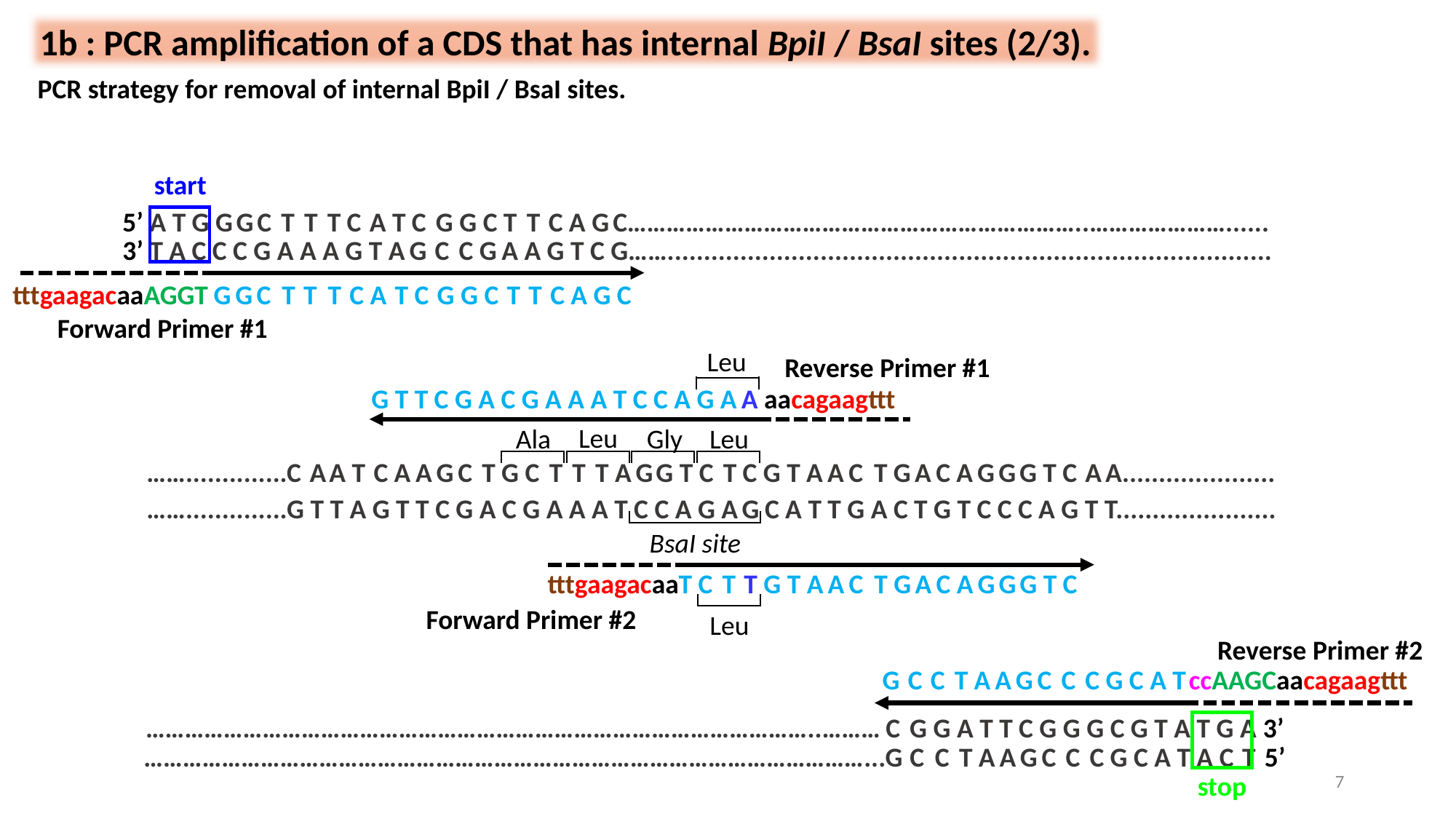

1b : PCR amplification of a CDS that has internal BpiI / BsaI sites (2/3).
PCR strategy for removal of internal BpiI / BsaI sites.
start
5’ A T G G G C T T T C A T C G G C T T C A G C……………………………………………………………..…………………......
3’ T A C C C G A A A G T A G C C G A A G T C G……...................................................................................
tttgaagacaaAGGT G G C T T T C A T C G G C T T C A G C
Forward Primer #1
Leu
Reverse Primer #1
G T T C G A C G A A A T C C A G A A aacagaagttt
Leu
Leu
Ala
Gly
……..............C A A T C A A G C T G C T T T A G G T C T C G T A A C T G A C A G G G T C A A.....................
……..............G T T A G T T C G A C G A A A T C C A G A G C A T T G A C T G T C C C A G T T......................
BsaI site
tttgaagacaaT C T T G T A A C T G A C A G G G T C
Forward Primer #2
Leu
Reverse Primer #2
G C C T A A G C C C G C A T ccAAGCaacagaagttt
…………………………………………………………………………………………..……… C G G A T T C G G G C G T A T G A 3’
…………………………………………………………………………………………………...G C C T A A G C C C G C A T A C T 5’
7
stop

## Slide 8
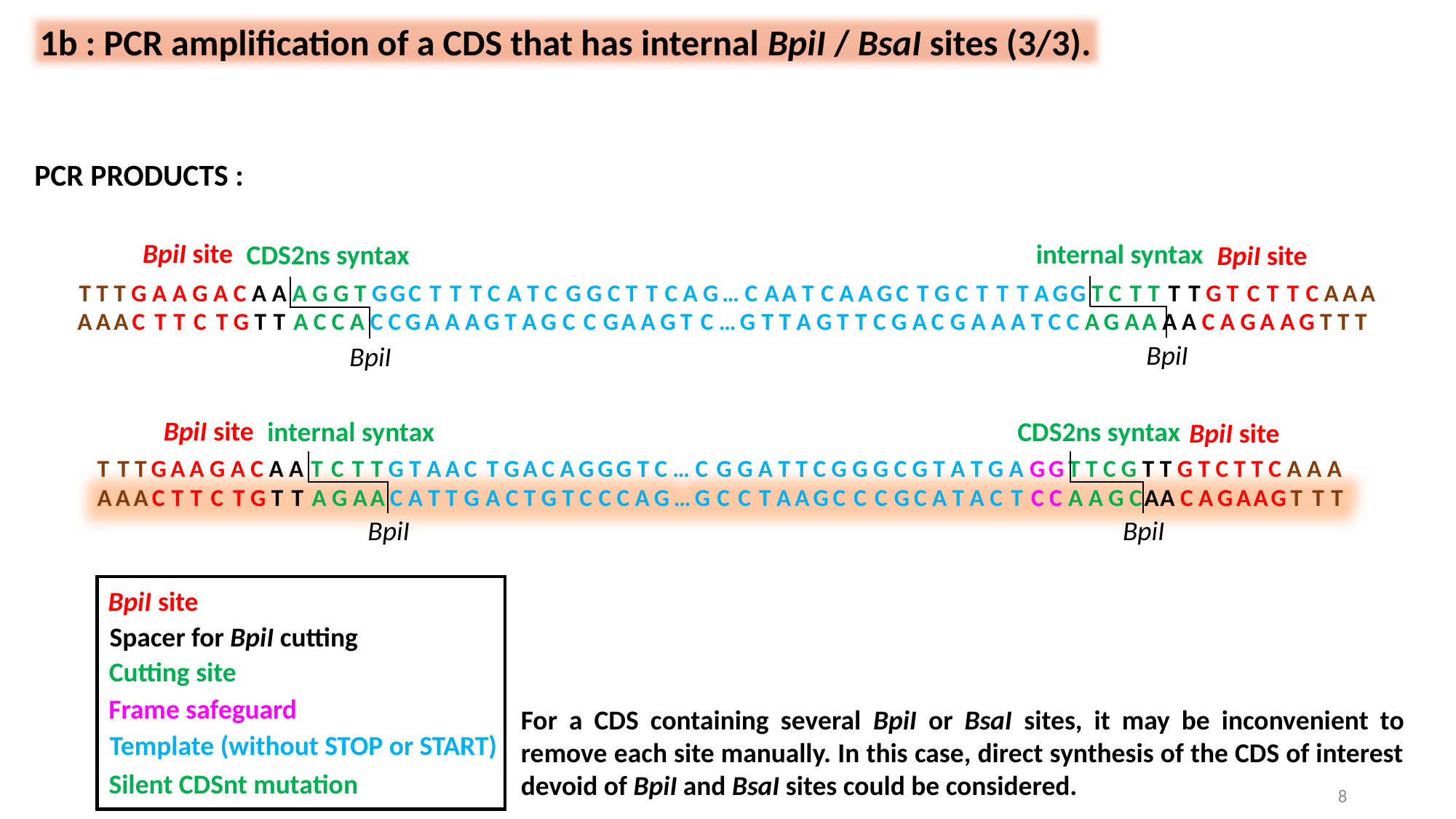

1b : PCR amplification of a CDS that has internal BpiI / BsaI sites (3/3).
PCR PRODUCTS :
BpiI site
internal syntax
CDS2ns syntax
BpiI site
T T T G A A G A C A A A G G T G G C T T T C A T C G G C T T C A G … C A A T C A A G C T G C T T T A G G T C T T T T G T C T T C A A A
A A A C T T C T G T T A C C A C C G A A A G T A G C C G A A G T C … G T T A G T T C G A C G A A A T C C A G A A A A C A G A A G T T T
BpiI
BpiI
BpiI site
CDS2ns syntax
internal syntax
BpiI site
T T T G A A G A C A A T C T T G T A A C T G A C A G G G T C … C G G A T T C G G G C G T A T G A G G T T C G T T G T C T T C A A A
A A A C T T C T G T T A G A A C A T T G A C T G T C C C A G … G C C T A A G C C C G C A T A C T C C A A G C A A C A G A A G T T T
BpiI
BpiI
BpiI site
Spacer for BpiI cutting
Cutting site
Frame safeguard
For a CDS containing several BpiI or BsaI sites, it may be inconvenient to remove each site manually. In this case, direct synthesis of the CDS of interest devoid of BpiI and BsaI sites could be considered.
Template (without STOP or START)
Silent CDSnt mutation
8

## Slide 9
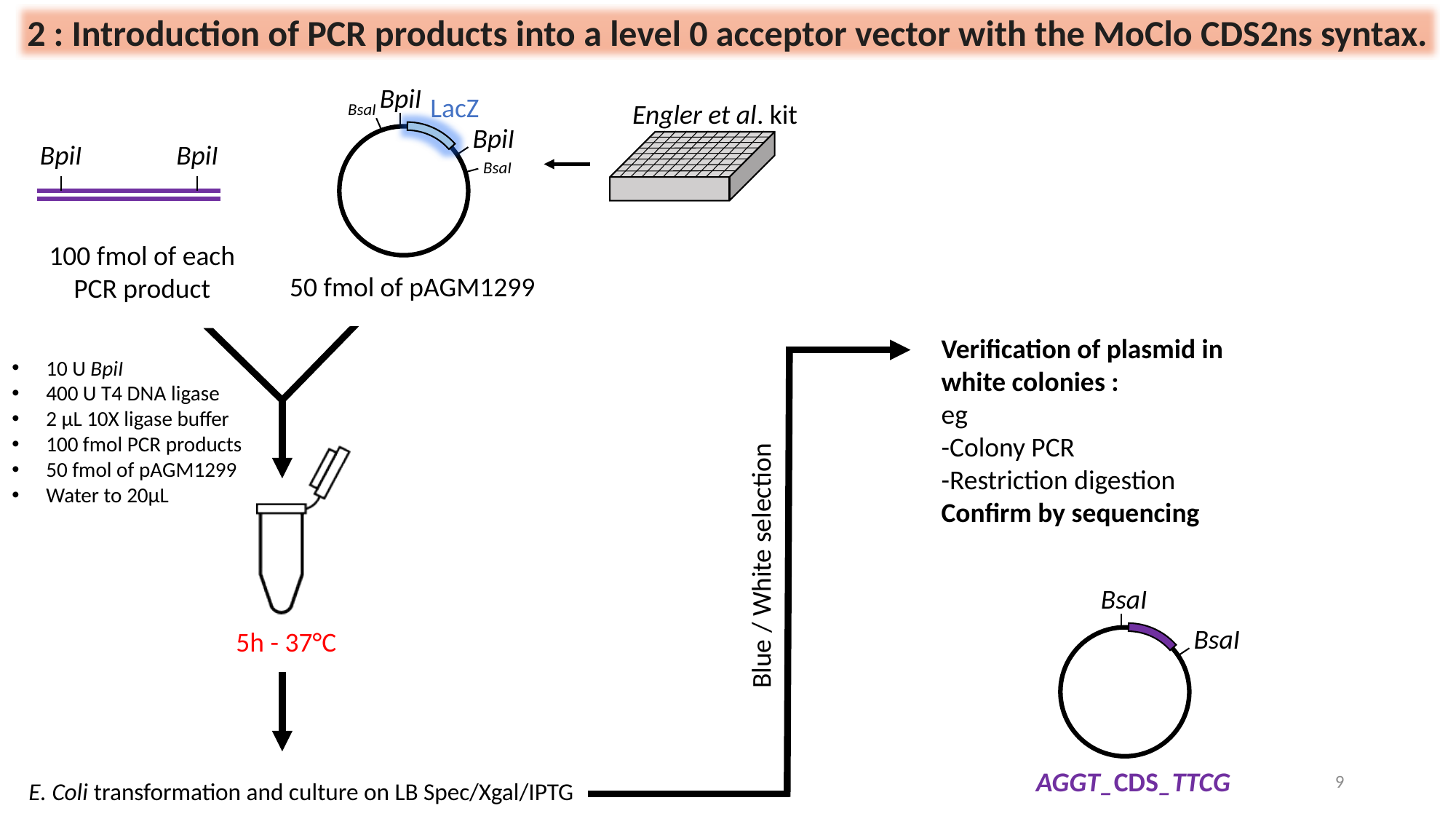

2 : Introduction of PCR products into a level 0 acceptor vector with the MoClo CDS2ns syntax.
BpiI
LacZ
Engler et al. kit
BsaI
BpiI
BpiI
BpiI
BsaI
100 fmol of each
PCR product
50 fmol of pAGM1299
Verification of plasmid in
white colonies :
eg
-Colony PCR
-Restriction digestion
Confirm by sequencing
10 U BpiI
400 U T4 DNA ligase
2 µL 10X ligase buffer
100 fmol PCR products
50 fmol of pAGM1299
Water to 20µL
Blue / White selection
BsaI
BsaI
5h - 37°C
AGGT_CDS_TTCG
9
E. Coli transformation and culture on LB Spec/Xgal/IPTG

## Slide 10
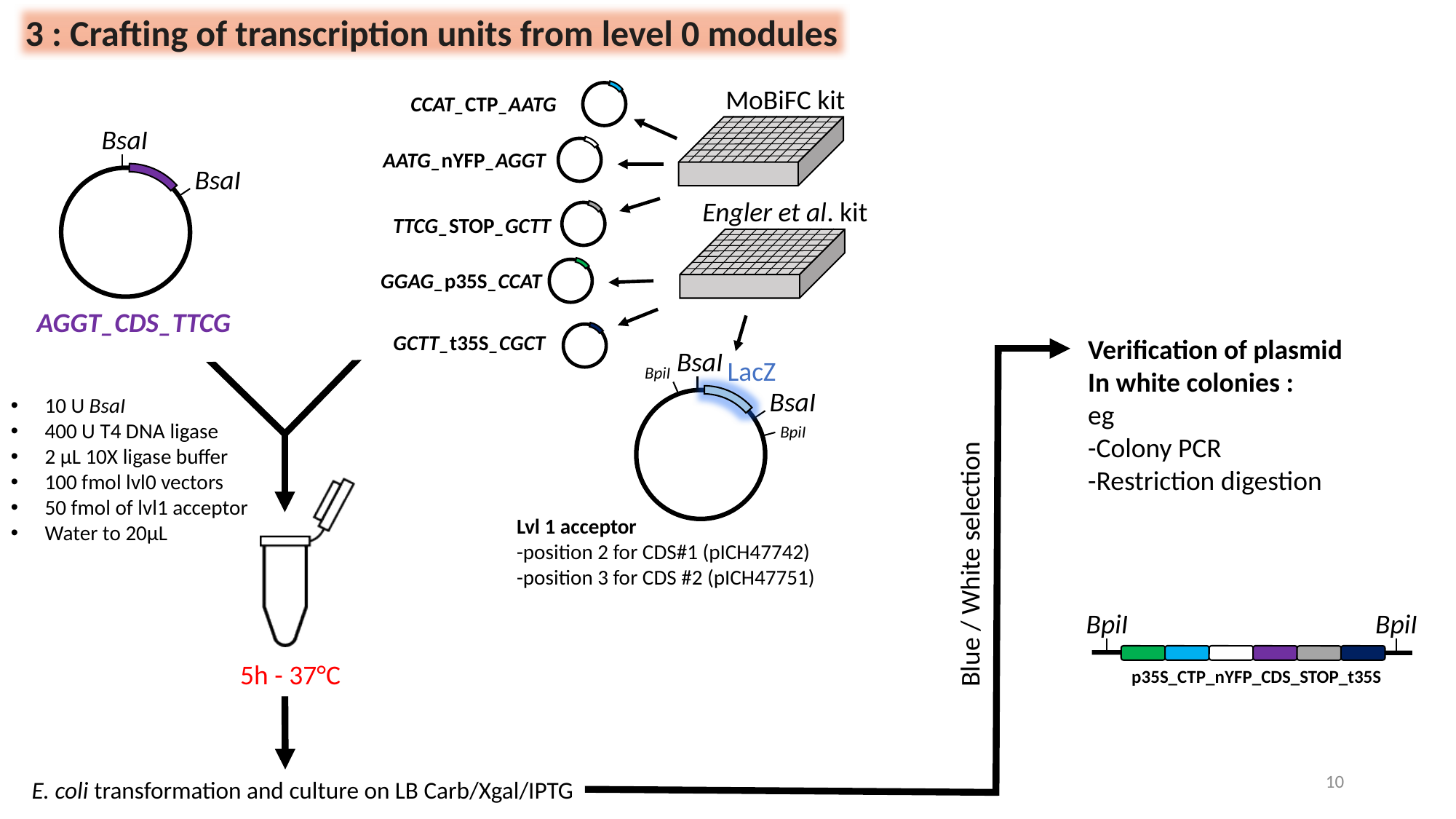

3 : Crafting of transcription units from level 0 modules
MoBiFC kit
CCAT_CTP_AATG
BsaI
AATG_nYFP_AGGT
BsaI
Engler et al. kit
TTCG_STOP_GCTT
GGAG_p35S_CCAT
AGGT_CDS_TTCG
GCTT_t35S_CGCT
Verification of plasmid
In white colonies :
eg
-Colony PCR
-Restriction digestion
BsaI
LacZ
BpiI
BsaI
BpiI
10 U BsaI
400 U T4 DNA ligase
2 µL 10X ligase buffer
100 fmol lvl0 vectors
50 fmol of lvl1 acceptor
Water to 20µL
Lvl 1 acceptor
-position 2 for CDS#1 (pICH47742)
-position 3 for CDS #2 (pICH47751)
Blue / White selection
BpiI
BpiI
5h - 37°C
p35S_CTP_nYFP_CDS_STOP_t35S
10
E. coli transformation and culture on LB Carb/Xgal/IPTG

## Slide 11
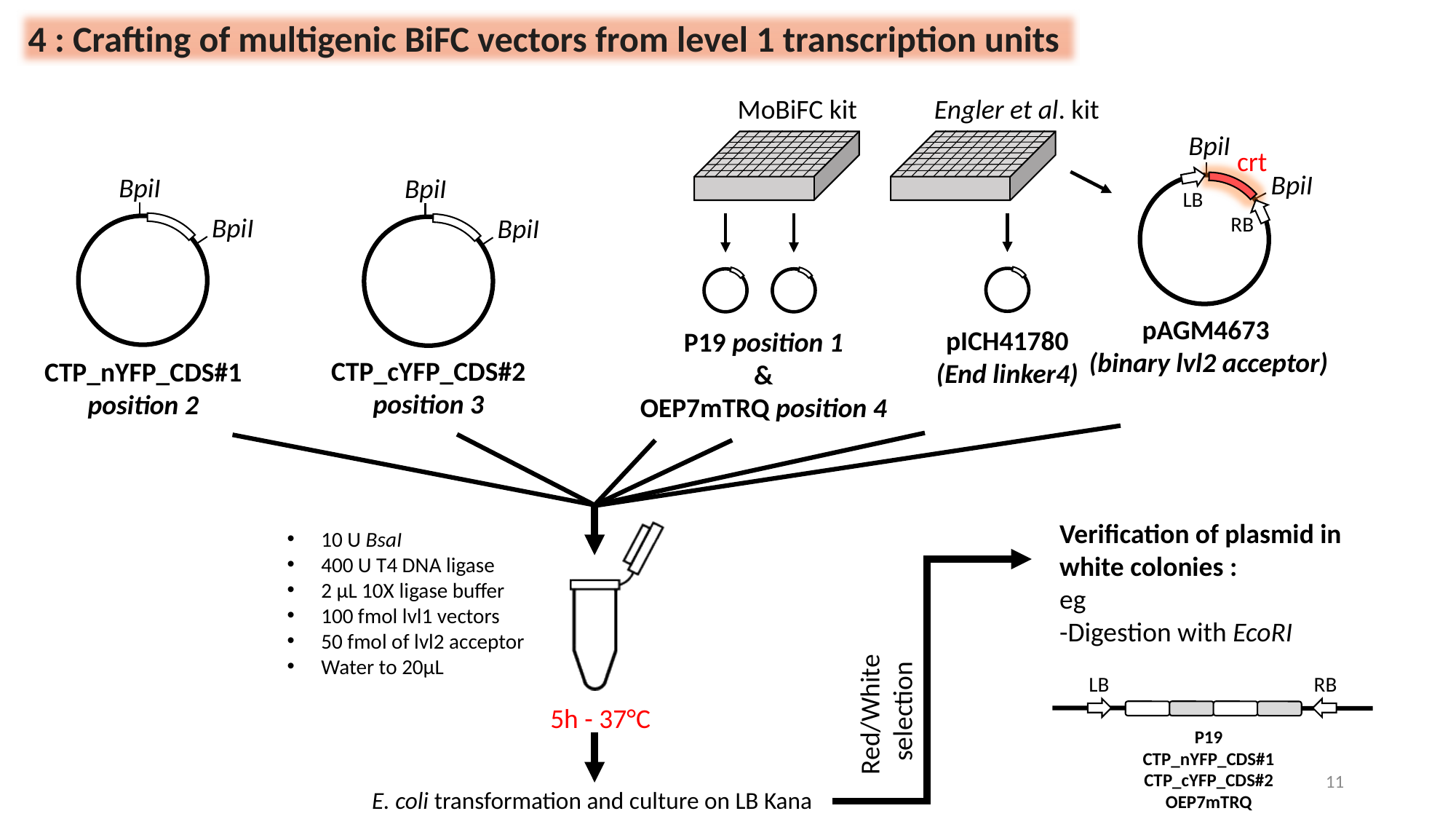

4 : Crafting of multigenic BiFC vectors from level 1 transcription units
MoBiFC kit
Engler et al. kit
BpiI
crt
BpiI
BpiI
BpiI
LB
BpiI
RB
BpiI
pAGM4673
(binary lvl2 acceptor)
pICH41780
(End linker4)
P19 position 1
&
OEP7mTRQ position 4
CTP_cYFP_CDS#2
position 3
CTP_nYFP_CDS#1
position 2
Verification of plasmid in
white colonies :
eg
-Digestion with EcoRI
10 U BsaI
400 U T4 DNA ligase
2 µL 10X ligase buffer
100 fmol lvl1 vectors
50 fmol of lvl2 acceptor
Water to 20µL
LB
RB
Red/White
selection
5h - 37°C
P19
CTP_nYFP_CDS#1
CTP_cYFP_CDS#2
OEP7mTRQ
11
E. coli transformation and culture on LB Kana
